# Adaptive coding for DNA storage with high storage density and low coverage

**DOI:** 10.1038/s41540-022-00233-w

**Published:** 2022-07-04

**Authors:** Ben Cao, Xiaokang Zhang, Shuang Cui, Qiang Zhang

**Affiliations:** School of Computer Science and Technology, Dalian University of Technology, 116024 Dalian, China

**Keywords:** Computational biology and bioinformatics, DNA computing and cryptography, Nanobiotechnology, Synthetic biology

## Abstract

The rapid development of information technology has generated substantial data, which urgently requires new storage media and storage methods. DNA, as a storage medium with high density, high durability, and ultra-long storage time characteristics, is promising as a potential solution. However, DNA storage is still in its infancy and suffers from low space utilization of DNA strands, high read coverage, and poor coding coupling. Therefore, in this work, an adaptive coding DNA storage system is proposed to use different coding schemes for different coding region locations, and the method of adaptively generating coding constraint thresholds is used to optimize at the system level to ensure the efficient operation of each link. Images, videos, and PDF files of size 698 KB were stored in DNA using adaptive coding algorithms. The data were sequenced and losslessly decoded into raw data. Compared with previous work, the DNA storage system implemented by adaptive coding proposed in this paper has high storage density and low read coverage, which promotes the development of carbon-based storage systems.

## Introduction

Massive data brings convenience and comes with great challenges at the same time. In light of this, how to use and store huge amounts of data wisely has become a difficult problem for data scientists. The International Data Corporation has predicted that the capacity of the global data will increase to 175 ZB by 2025. Modern storage systems face the problems of high cost and high energy consumption, and DNA has captured the attention of researchers as a storage medium with high parallelism, low maintenance cost, and great storage potential. DNA also offers many other unique potential advantages, such as decades or centuries of stability compared with traditional media that are updated every few years and easy replication based on molecular biology methods to prevent degradation.

**Fig. 1:**
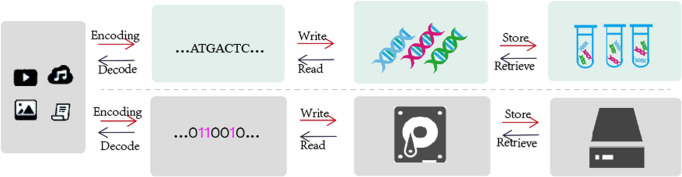
DNA storage versus conventional magnetic storage.

DNA storage refers to the use of DNA bases to store data, and the schematic diagram of traditional magnetic storage is shown in Fig. 1. The first attempts to use DNA to store abiotic information date back to the late 20th century, when Joe Davis^1^ pioneered the use of bacteria as a storage medium for abiotic information. However, biotechnologies such as synthesis and sequencing were limited at that time, and the field did not develop rapidly. In 2001, Bancroft et al.^2^ encoded two quotes from the opening lines of A Tale of Two Cities into a DNA molecule using a method similar to the codon method used in DNA to encode protein sequences. In 2012, Church et al.^3^ encoded text, JavaScript programs, and images into corresponding DNA sequences that were eventually deposited in DNA and could handle errors from DNA sequencing and synthesis. Birney et al.^4^ took a 26-second “I Have a Dream” speech fragment into a DNA sequence and stored it in DNA after complete synthesis. In 2015, Yazdi et al.^5^ proposed an efficient storage architecture that appended specific unique address bits of 20 bps length to the ends of a 1000 bps data block to store the encoded Wikipedia of six universities. Furthermore, Organick et al.^6^ in 2018, proposed an end-to-end DNA data storage that implemented 200 Mb of data in DNA, demonstrating large-scale, random-access capabilities. In 2019, Lee et al.^7^ described a de novo synthesis strategy for DNA data storage that utilized template-independent polymerase terminal deoxynucleotidyl transferase under motion-controlled conditions. In 2020, Bee et al.^8^ proposed a molecular-level similarity search method comparable to the state-of-the-art in silico similarity search algorithms that demonstrated a technique to perform similarity search on a DNA-based database of 1.6 million images. Natural DNA molecules contain four bases and can store up to 2 bits of information per base^9–13^.

The first step in storing information in conventional magnetic storage media is to efficiently encode the information to be stored; this is also true in DNA storage, where encoding is the top priority. A reasonable and efficient encoding of DNA not only reduces coverage and improves base utilization but also reduces errors, improves storage system coupling, and maintains data integrity. The error-prone stages in DNA storage are mainly during synthesis and sequencing, where most of the errors come from the sequencing process again. Moreover, rational and efficient coding can improve the accuracy rate of sequencing results and thus reduce the number of repeat sequencing cycles. Therefore, the coding problem in DNA storage has received a lot of attention from researchers.

Huffman coding is a widely used coding method for data file compression, commonly used for lossless data compression with compression ratios of 20% to 90%. In 2013, Goldman et al.^4^ proposed an encoding scheme using Huffman coding to increase the coding potential in DNA storage to 1.58 bits/nt. Then, Grass et al.^14^ using Galois field (GF) and Reed–Solomon (RS) codes to correct storage-related errors. In 2016, Bornholt et al.^15^ used the XOR encoding code with Goldman’s encoding scheme and proposed a DNA-based system. Fountain code is a widely used information encoding method in communication systems. In 2017, Erlich and Zielinski^16^ proposed DNA storage based on the fountain code encoding scheme, which prevented single-nucleotide duplication and GC content anomalies. Both Jeong^17^ and Anavy^18^ made further improvements to Erlich’s initiative, reducing the read cost by 6.5%–8.9% in Jeong’s initiative, and increasing storage density by 24% in Anavy’s initiative. In addition to the use of fountain codes to reduce the generation of errors in DNA storage, Immink et al.^19^ described a simple and efficient implementation of coding techniques to avoid the appearance of long homopolymers. Yazdi et al.^20^ used weak mutual uncorrelation (WMU) coding for primer design DNA storage coding. Song et al. proposed a method for converting 0–1 sequences into DNA base sequences that encoded results satisfying the runlength constraint and GC-content constraint and achieved a rate of 1.90 bits/nt with low encoding/decoding complexity and limited error propagation. Error correction coding satisfying constraints^21–25^ and focusing to improve storage density^26^ have also received extensive attention. 2021, Zhu et al.^27^ used quaternary barcodes to encode images into 16 DNA fragments, and eventually, images were successfully preserved, encrypted, and recovered while avoiding any protein or enzyme reactions. This work implemented a nanopore platform for DNA storage systems with improved capacity and programmability. In addition, there are also related DNA storage schemes for reliable and orthogonal information encoding in living cells^28^.

DNA as a storage medium has a high theoretical storage density, but it is difficult to reach the theoretical limit due to errors inherent in DNA storage channels (sequencing and synthesis errors) and biochemical limitations of sequences^29^. Therefore, much work has focused on optimizing coding methods to reduce information redundancy, such as Huffman codes and RS codes^30,31^. Importantly, each DNA sequence, in addition to encoding payload bits (data bits)^32,33^, needs to contain associated primers, address bits, and other non-payload bits, which are effective safeguards for the integrity of DNA stored data. The ability to selectively access only part of the information is necessary to make DNA storage viable. Implementing random access has become the focus of current researchers^34^. Widely used methods for random access are address bit addressing^31^ and magnetic bead extraction methods^35^. Different types of stored information, different environments, and different methods lead to differences in DNA storage system models. Although researchers have proposed various DNA storage coding schemes with different characteristics and preliminarily verified the coding potential of DNA storage and the possibility of replacing silicon-based storage to some extent, the recently proposed DNA storage systems still have certain problems: (1) DNA as a storage medium has high theoretical storage density, but the storage density of existing DNA storage systems is far from the theoretical value. (2) DNA storage often generates errors during synthesis and sequencing, and redundant information, such as error correction, needs to be added; alternatively, sequencing coverage and double-end reads must be increased in sequencing to maintain data consistency; all of this increases sequencing coverage. (3) Previous work has effectively advanced coding at various locations but lacks a unified approach from the system level to deal with different coding location coordination errors, the lack of connection between the encoding results of different locations, and the poor overall coupling of the system.

Although the field of DNA storage research has been steadily developing in recent years, a DNA storage system with high utilization of DNA sequences, low read coverage, and high coupling between different coding locations is lacking. Therefore, in this paper, we propose an adaptive coding DNA storage system with high storage density and low coverage and with high coupling between different coding locations using different coding schemes for different functional areas. We optimize the system at the hierarchical level to ensure the efficient operation of each link. First, for payload bits, the storage files are converted to binary by corresponding conversion methods; continuity, GC content, and base balance degree constraint are satisfied, and parameters such as GC content are calculated for non-payload coding constraint thresholds after coding is completed. Second, the non-payload bits are constructed using an intelligent optimization algorithm under the condition of satisfying the combination constraint, where the encoding constraint threshold of the non-payload is generated adaptively from the encoding result of the payload. The payload and non-payload are assembled with high coupling. Then, in the decoding stage of the read information, a certain number of reads are sampled from the read results of each simulated sequencing of the resultant FASTQ file. Finally, these reads are merged using the sequence collocation algorithm, and the raw data are finally obtained using the combined FASTQ file for RS-corrected LT decoding. The adaptive coding DNA storage system proposed in this paper has achieved satisfactory results in storage density, storage capacity, support for random storage, and coverage.

## Result

**Fig. 2:**
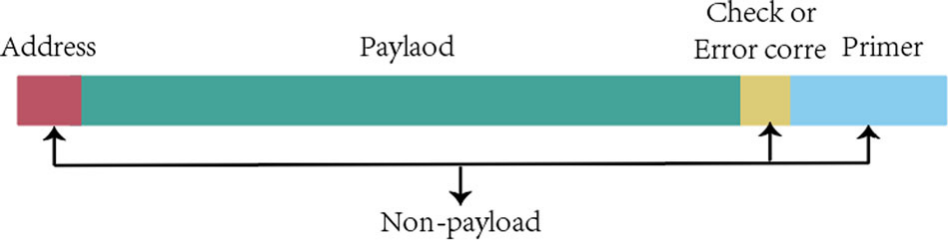
Different coding classes in DNA storage.

Low storage density, high sequencing coverage, and low coding coupling are common problems in DNA storage. The coupling degree is very important in DNA storage coding, because payload and non-payload bits need to be encoded separately according to different conditions and constraints in DNA storage, ensuring the coupling degree between different coding locations can ensure the robustness of the final synthesized DNA sequence. For example, if there is payload bits A and non-payload bits B, respectively, encoded to satisfy the constraint, then both A and B satisfy the constraint, but when A and B assemble into the DNA sequence to be synthesized, the last base of B and the first base of A may be the same, which breaks no-runlength constraint, reduces the overall robustness. In this case, the coupling degree of coding is low, because in the process of coding, the connection between payload and non-payload bit coding is poor, and there is no comprehensive consideration. Of course, this example is only in the simplest case, where the DNA storage system is much more complicated. In this section, we show that the adaptive coding DNA storage system proposed in this paper can reduce the impact of common problems on system performance by comparing the base distribution, the assembly of DNA coding, and the DNA storage results. The distribution of base functional bits on DNA sequence in the adaptive coding of DNA storage system is shown in Fig. 2. First, by comparing the base distribution, it is illustrated whether the encoding result of the payload is a high base balance library, which is crucial to obtain higher-quality sequencing data^36^, reduce the coverage, and ensure the effectiveness of the base balance constraint. Then, the comparison of the assembly results illustrates whether adaptive coding during the assembly process improves the coupling of different coding positions. Finally, the adaptive DNA storage system is compared with previous work in terms of storage density, storage capacity, and sequencing coverage, and an independent random-access experiment is performed to verify the performance of the storage system.

### Contrast the distribution of bases

**Fig. 3:**
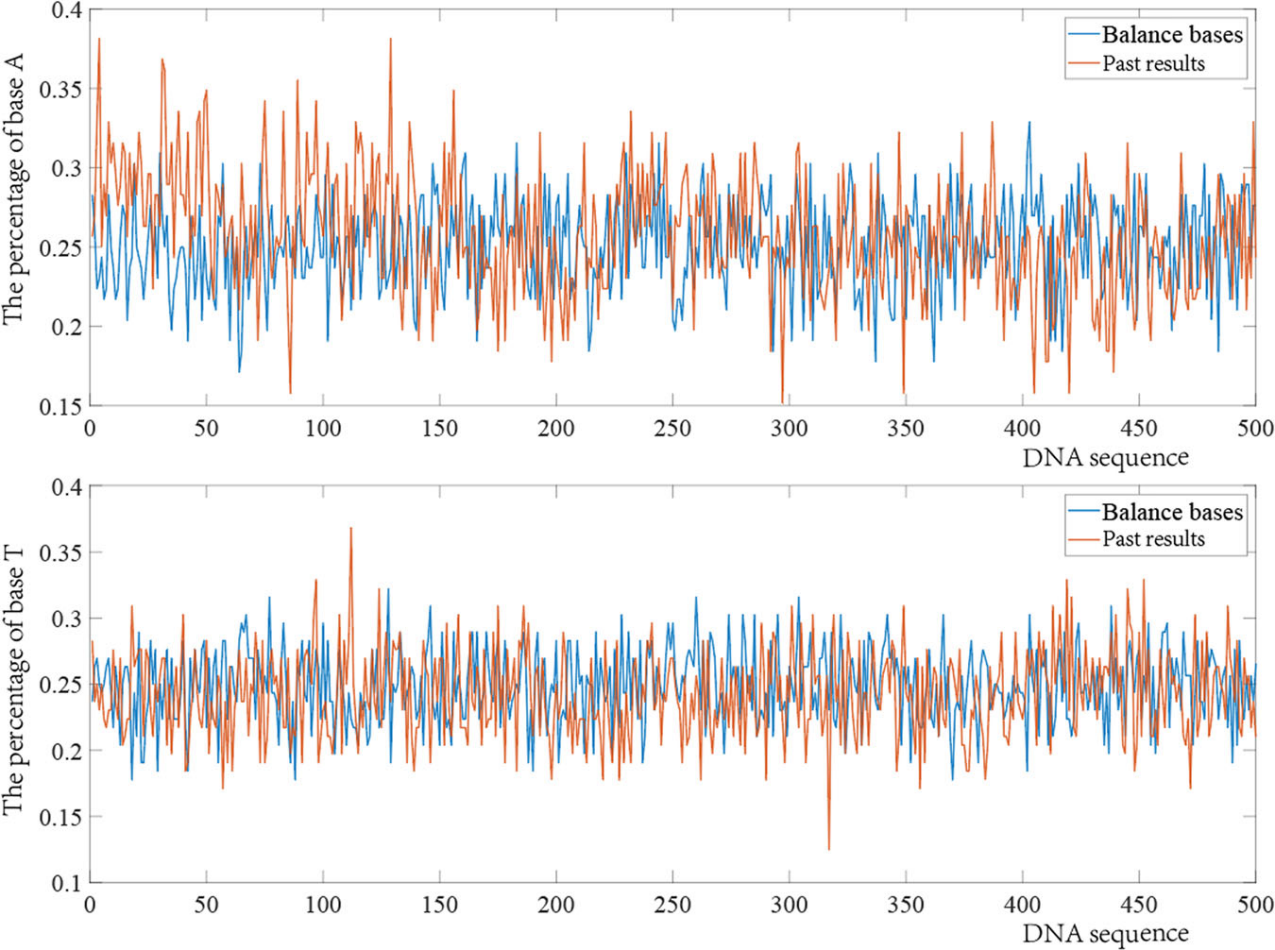
The influence of high base balance constraint on A and T base contents.

**Fig. 4:**
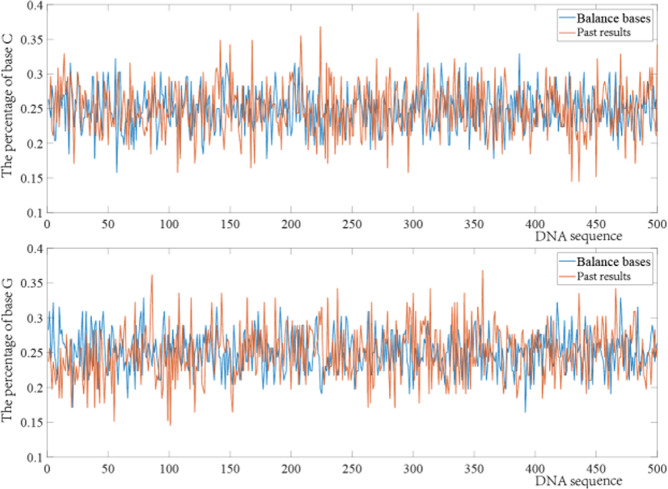
The influence of high base balance constraint on C and G base contents.

To verify the effectiveness of the base balance constraint, the result plots of the effect of adding the base balance constraint on the distribution of bases in the coding results are shown in Figs. 3 and 4, where the blue lines represent the results of the coding in this paper and the orange lines represent the results of Jeong et al.^17^. In order to get a clearer view of the different base balances in the figure, the notes for balanced bases have been bolded. It is clear from the fluctuation of the lines and the maximum and minimum values that the payload coding in this paper has a better base balance in DNA sequencing, and Illuminate’s sequencing manual^36^ states that balancing the library allows the sequencing system to keep focus better and deliver higher-quality data.

In addition, to more effectively illustrate the good base balance in the proposed payload coding scheme, a random sampling of all coding results was also performed, with the same number of bases taken each time. The sum of variances, means, and medians of the sampled base fragments are compared in Table 1, and compares the work in this paper with the original coding scheme of Jeong et al.^17^ and the results of Jeong with the base balance constraint. The detailed comparison schemes are shown in Supplementary file 1. The smaller the sum of variance, mean, and median, the higher the base balance, and the smaller the bias of sequencing can improve data quality^37^, which in turn reduces the number and coverage of sequencing. There are two reasons why the base distribution in the DNA sequence is more ideal in the results of adaptive coding. First, the adaptive coding considers both payload and non-payload sites, which improves the coupling of the overall coding. Second, compared with Jeong et al.’s work^17^, GC content constraint is simply considered in data bits. However, when the sequence is longer, GC content is satisfied as a whole, but some DNA blocks may have large GC deviations.

**Table 1:** Sum of variances, mean, and median comparisons of fragments of length 100 bases.

	Sum of the variance	Mean	Median
Jeong^17^	4.35E + 06	241.7865	2.32E + 02
Improve Jeong	3.41E + 06	189.3271	1.76E + 02
In this work	3.26E + 06	181.3739	1.77E + 02

### Comparison of coding integrity

**Fig. 5:**
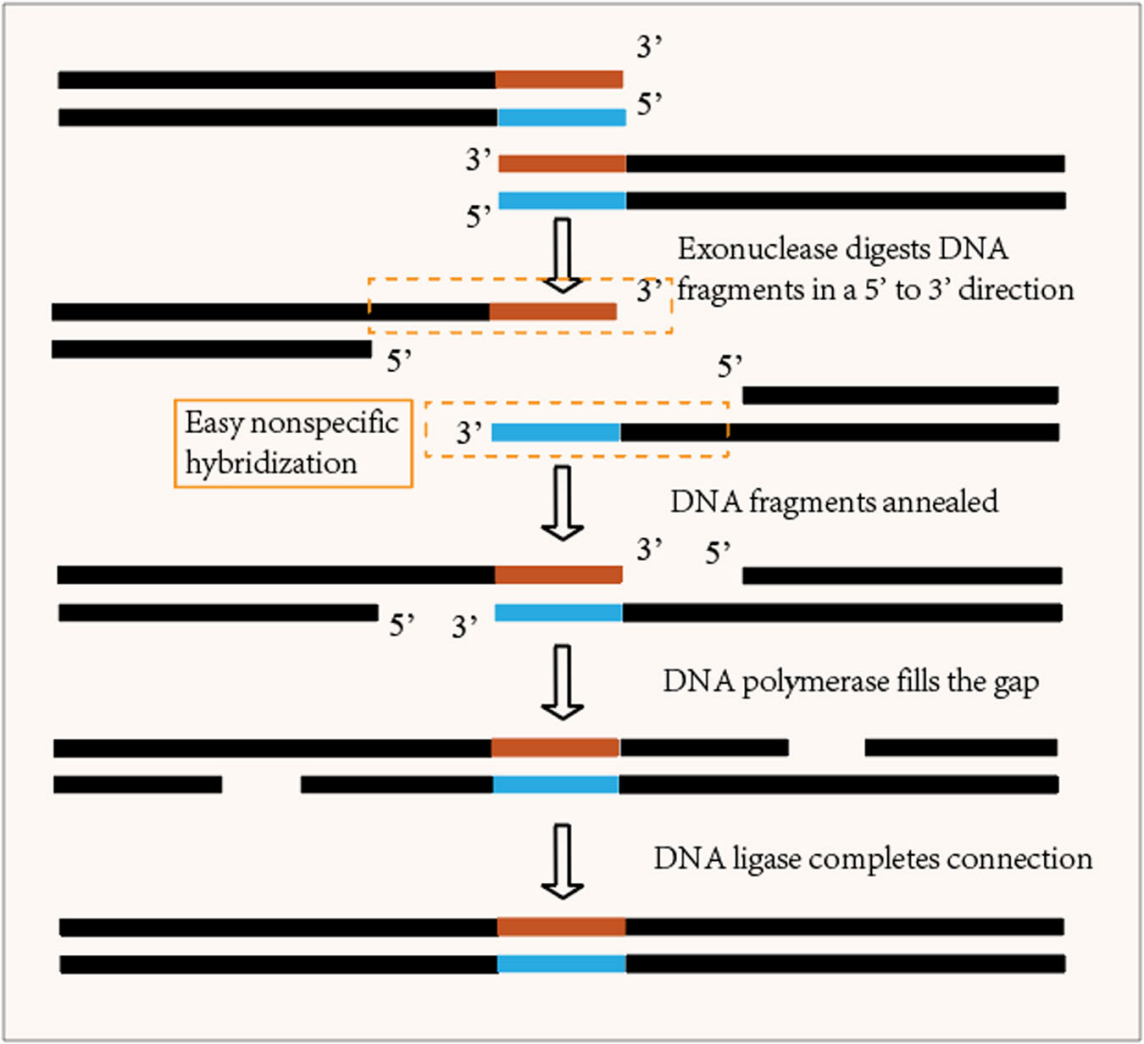
Gibson assembles address bits and data bits.

To illustrate that the adaptive coding algorithm can reduce the problem of poor coupling between different coding positions in previous work, the integrity of coding is compared in this section. The coupling of address bit and data bit coding were evaluated by the stability and thermodynamic properties of bare DNA single strands during assembly. When the coding of each part is completed and DNA synthesis is performed, the assembly of the coding results of different parts of the DNA sequence is required. In most previous works, no attention has been paid to increasing the coupling between the payload and non-payload. In contrast, in this paper, the coupling is improved and collisions between primers and payloads are reduced by efficient screening and adaptive coding of address and data bits. In the Gibson assembly process in Fig. 5, the use of the nucleic acid exonuclease to ablate the 5′−3′ is shown. The process of ablating the 5′−3′ strand using the nuclease results in the exposure of the complementary strand; thus, the evaluation of the coupling between the address and data bits can be accomplished by measuring the stability and thermodynamic properties of this segment of DNA.

To reflect the merit of the assembly results, Table 2 presents a comparison of the performance of the exposed single-strand during the assembly process in the work of Jeong et al.^17^ and that of this paper. Biochemical reactions are involved in DNA unchain and synthesis, and the essence of biochemical reactions is a change in energy. The size of minimum free energy (MFE) can reflect the stability of DNA single chain and the error rate in PCR and sequencing to a certain extent^38^. Therefore, this paper illustrates the advantages of coding algorithm by comparing MFE. MFEAVE, MFEMAX, and MFEMIN represent the mean, maximum, and minimum values of the minimum free energy, respectively, and TM variance represents the variance of the melting temperature (TM). The results encoded in the work of Jeong et al. are compared with the four representative schemes in this paper. Smaller TM variance and MFE can indicate stronger sequence stability, the full names of the specific combined constraint schemes are given in Table 3. By comparing the MFE and TM variances, it is clear that the assembly results in this paper have good stability and thermodynamic properties that reduce the generation of read and write errors. MFEAVE and TM variances were reduced by 21–26% and 10%, respectively. A smaller MFE free energy indicates that the single strand of DNA is more stable in solution, and a smaller TM variance indicates that the TM of DNA sequences is more similar in PCR and other processes, resulting in fewer errors. DNA coding is superior to Jeong in expected attributes (GC balance, MFE, continuous base, etc.), which can reduce error rate bias in the sequencing process^37^. A higher-quality coding assembly strategy can improve intercoding coupling, which has the effect of avoiding additive-memory crosstalk, reducing the number of repeat sequencings, and decreasing coverage. In addition, a comparison of the thermodynamics and stability of the assembly schemes under different constraints of the same length is also presented in Supplementary Tables 1–3, further indicating the superiority of the adaptive coding proposed in this work.

**Table 2:** Comparison of single-strand MFE and TM.

	MFEAVE	MFEMAX	MFEMIN	TM variance
Jeong^17^	−11.5095	−4.9	−26.3	2.2409
HGN	−14.238	−3.4	−23.9	2.034
HGNN	−14.0197	**−5.6**	−26.7	2.0542
EGNA	**−14.4701**	−4.8	−31.1	2.0271
EGNAM	−14.4146	−5.0	**−32.7**	**2.027**

**Table 3:** Constraint coding condition abbreviation and full name contrast.

Acronym	Full name
HGN	Hamming distance constraint, GC-content constraint, No-runlength constraint
HGNN	Hamming distance constraint, GC-content constraint, No-runlength constraint, Non-adjacent subsequence
EGNA	Storage edit distance constraint, GC-content constraint, No-runlength constraint, Uncorrelated of the address constraint,
EGNAM	Storage edit distance constraint, GC-content constraint, No-runlength constraint, Uncorrelated of the address constraint, Minimum free energy (MFE) constraint

### Storage integrity comparison

After completing data writing and reading, to verify the performance of the proposed adaptive encoding DNA storage system, comparisons were made with the previous representative works in various aspects, namely storage density, coverage, storage capacity, and encoding methods (Table 4). The storage density, storage capacity, and coverage in the table respectively refer to the data storage capacity of each base, the size of data stored, and the coverage degree of DNA sequence in the sequencing process. It is worth mentioning that the increase in coverage in the sequencing process will make it difficult to read information. The calculation method and explanation of the different storage densities in the table are in supplementary document 1.

**Table 4:** Comparison of an adaptive coding DNA storage system with previous work.

Refs.	Length (nt)	Bits per base including primers	Bits per base excluding primers	Random access	Coverage	Code	Contents	Storage capacity
Church^3^	115	0.60	0.83	No	3000×	1 bit to 1 base	English text, JPG images, computer code	650 KB/630 KB
Goldman^4^	117	0.19	0.33	No	51×	Rotating encoding	Text file, JPEG file, MP3 file	739 KB
Grass^14^	158	0.86	1.14	No	372×	Reed–Solomon coding	Text from the Swiss Federal Charter	83 KB
Organick^29^	150–200	0.81	1.10	Yes	4–11×	Reed–Solomon coding	high-definition video, images, audio, and text	200.2 MB/33 KB
Bornholt^42^	120	0.57	0.85	Yes	40×	rotating encoding	Three JPG files	151 KB
Erlich^16^	152	1.18	1.57	No	10.5×	DNA fountain encoding	Text file, SVG file, Video file	2 MB
Jeong^17^	152	1.17	1.53	No	600×	DNA fountain encoding	JPG file	513.6 KB
Blawat^43^	230	0.89	1.08	No	160×	6 bis to 3 bases	MPEG compressed movie sequence	22 MB
Choi^26^	85	1.78	3.37	No	250×	One character	Text file	854 B
Yazdi^44^	880–1000	1.71	1.74	Yes	200×	14 bits to 8 bases	Two JPEG images	3 KB
Lee^7^	150–200	1.57	1.57	No	175×	2 bits to 1 base	Text message	18 B
This work	162	1.29/1.22	1.41	Yes	35×	DNA constraint + fountain encoding+RS	jifi, mp4, txt, jpg, pdf files	480 KB/83.3 KB

As can be seen in Table 4, the table is drawn in reference^6,31^, the adaptive coding DNA storage system proposed in this paper has excellent storage density even at low coverage, and the adaptive process adaptively generates thresholds for the coding constraints of the non-payloads from payload bits, which improves the coding coupling and increases the storage density. Due to the difficulty of synthesizing longer DNA strands, this scheme continues to follow DNA sequences with a length of ~100. In terms of storage density, the adaptive coding DNA storage system proposed in this paper reaches 1.29 bits/nt (if the file size is calculated before compression, the storage density can reach 1.87 bit/nt), which exceeds the work of Church et al. and stores more information using fewer bases. Although there is still a gap between choi et al.’s work, but the proposed method can support random access without any external memory. The sequencing coverage of the DNA storage system plays a decisive role in the complexity of information reading, and higher sequencing coverage makes it more difficult to read information. As shown in Table 4, the coverage of the storage system proposed in this paper is second only to that proposed by Erlich et al. and even an order of magnitude lower compared to other schemes. Although the adaptive coding DNA storage system is slightly weaker than that in Choi et al.’s work in terms of storage density, it is slightly higher than that of Erlich et al. in terms of sequencing coverage. However, a complete storage system needs to implement random access to information to reduce the difficulty of reading. Compared with the respective storage schemes of Choi and Erlich, this storage system can implement random access to data. Furthermore, this scheme stores 480 KB (Compression before 689 KB) of data, including Jifi, MP4, and TXT. Compared with Bornholt and Choi et al.’s simple storage of pictures and texts, this scheme has higher applicability and practical significance and is suitable for storing various forms of files in the information age. Although the storage capacity in this paper is still far behind that of Blawat and Organick, the adaptive coding DNA storage system has the characteristics of low coverage and reducing address–memory crosstalk. Moreover, compared to previous work in simple mapping coding, this paper uses adaptive coding with better coordination; the payload bits are coded using fountain codes; RS error correction codes are included; the non-payload bits are coded using constraint coding; the coding constraint thresholds of the non-payload bits are generated by the payload bits adaptively, which improves the overall coupling of the system.

It is important to note that in Table 4, we give two values for both storage density and storage capacity. The one on the left is the storage density for storing 480 KB data without rigor storing the addressing table, which is accepted by most existing work. The two mainstream storage density calculation methods include whether to include primers or not^6^. For rigorous consideration, the storage density calculation in this paper includes primers. The two storage density values in the table represent the storage density for 480 KB data (without storage addressing table) and the storage density for independent random storage Harry Potter 1 (including storage addressing table). Although compared with the calculation method without primers, the storage density on the left is already very convincing. However, in a storage system that supports random access, the address table needed to access data is often stored in external memory. Therefore, in order to better illustrate the performance of the adaptive scheme proposed in this paper under extreme conditions, such as earthquake, tsunami, electromagnetic storm, and other harsh conditions, or when the addressing table is damaged. Rigorous random-access experiments were also carried out, with excerpts from Harry Potter 1 stored in index pools and archive pools, finally achieving access requirements to read any paragraph. This method does not require any external memory, is truly random access, and the storage density is recorded to the right of the slash line in Table 4. Although the development of DNA storage is just beginning, especially for random access storage, the development of DNA storage is well promoted by strict standard random access.

## Conclusion

This paper reviews the development and architecture of DNA storage systems, highlights their advantages and disadvantages, describes the existing DNA storage steps in detail, and illustrates the technical details and problems of these steps. To address the problems of low storage density, high sequencing coverage, and poor coding integrity of traditional DNA storage systems, an adaptive coding DNA storage system with high storage density, low coverage, high base balance, and high coupling is proposed. The DNA storage system proposed in this paper has two stages. The writing of data is mainly encoded and then synthesized into DNA sequences by DNA synthesis technology and stored in DNA pools, and the reading of data is decoded after DNA sequencing. Encoding the data is the first and most important step in DNA storage. In this paper, different characteristics of the payload and non-payload are encoded separately. Payloads are encoded using a fountain code encoding scheme that satisfies constraints, homopolymer, GC content, and base balance degree constraints are imposed to guarantee the quality of the encoding. Base balance is essential for optimal run performance and high-quality data generation, it also reduces sequencing coverage during data reads. Compared to the coding results without added base balancing constraints, a higher base balance in each cycle can be seen in Figs. 3 and 4. For the encoding of the non-payloads using heuristic class algorithms constructed under combinatorial constraints, the thresholds of combinatorial constraints can be generated adaptively based on the encoding results of the payloads. In addition to the original combination method, this paper also gives other methods in Table 2. Additionally, the types of heuristic class algorithms and constraints can be selected according to different storage environments. By analyzing the stability and thermodynamic properties of the bare single strand during Gibson assembly, the advantages in dynamically modulating the non-payload by the characteristics of the payload are demonstrated, with a 21–26% and 10% reduction in MFEAVE and TM variance compared to previous work. A smaller MFE in the solution means a more stable single strand of DNA. The smaller variance of TM means that the TMs of DNA sequences in PCR and other processes are more similar, resulting in fewer errors. Furthermore, high-quality coding results not only increase storage density but also reduce the error rate, thereby affecting the sequencing coverage when the data are read. The experimental results show that files of images, videos, and PDFs of size 689 KB were successfully stored in DNA and eventually read out losslessly using the sequencing software. Further comparisons with previous work were made in the various aspects of storage density, coverage, storage capacity, and encoding methods. Except for slightly weaker performance than Erlich and Organick in terms of storage capacity and coverage, ideal results were achieved in all other cases; in terms of random storage potential and storage density, the adaptive encoding DNA storage system proposed in this paper provides great advantages compared with previous work. In order to better explain the random access function of the adaptive DNA storage system proposed in this paper, an independent random storage test was carried out, and finally a section of Harry Potter 1 was read.

In future work, we will implement an adaptive encoding DNA storage system for sequencing coverage, storage density, storage capacity, and other storage-related indexes with better performance and features to improve the storage density and capacity and minimize the coverage to reduce the cost. However, wet experiments have not been conducted in this study. Therefore, we will continue to promote the practical application of DNA storage in our future work and carry out related work in terms of both storage cost and synthetic biology development to improve the storage potential of DNA storage and the availability of cold data.

## Methods

**Fig. 6:**
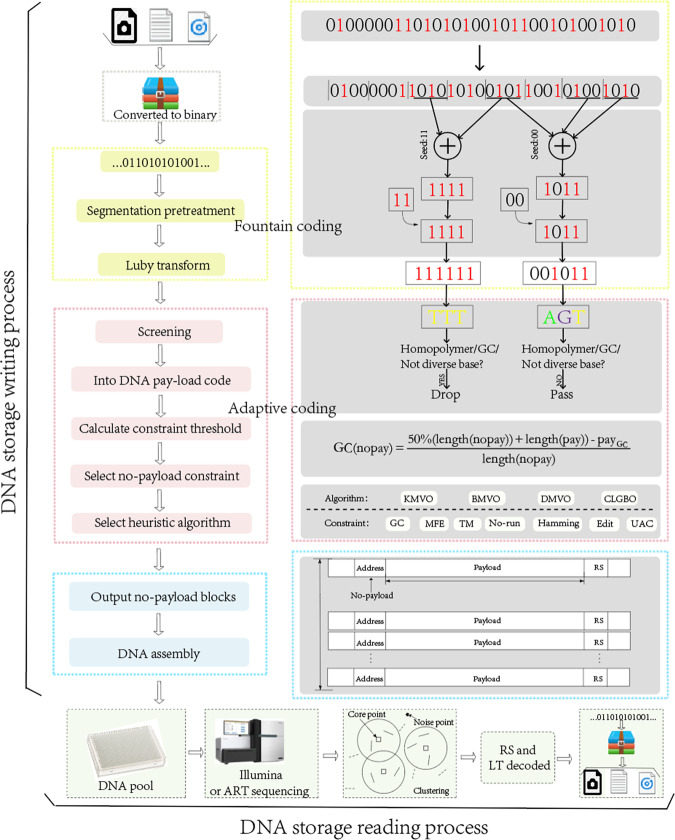
Process diagram of an adaptive coding for DNA storage system.

After transforming the original file into binary, the adaptive coding DNA storage system uses different coding methods for two different cases of payload and non-payload depending on the characteristics of the coding location. First, the fountain code that meets not only the homopolymer and GC content constraints, but also the base balance degree constraint is used for payload encoding. Then, for non-payload coding processes, a strategy of constraint filtering is used, and a variety of algorithms and combined constraints are provided to choose from. This paper not only provides the combination of constraints that appeared in the past work, but also the repetition of constraint candidate solutions would cause a waste of computing power, so it is further extended. In this paper, DMVO algorithm and HGN combination constraints are selected according to the size of stored files and storage scenarios. And the constraint threshold of non-payload, such as the range of GC content and the threshold of homopolymer, is generated adaptively by the characteristics of payload. Finally, the DNA sequences in the DNA pool are sequenced using sequencing software^39^, and the original data are restored using the sequence collocation algorithm and the RS error correction algorithm. A schematic diagram of the full process of the adaptive coding DNA storage system is given in Fig. 6. In this section, first, the base balance constraint is introduced. A high base balance library can provide high-quality sequencing data to reduce the coverage. Then, the adaptive coding threshold method is proposed to improve the coupling between different coding positions, and finally, a more convincing independent random access experiment is performed.

In the coding process, some coding constraints are proposed for different error causes to ensure the coding quality, such as Hamming distance and store edit distance constraint^32^, minimum free energy constraint^40^, No-runlength constraint^41^. The Hamming distance and store edit distance constraints are designed to reduce the similarity between DNA sequences and avoid nonspecific hybridization. The minimum free energy constraint screens DNA code words with more stable thermodynamic properties based on the heat variation in the reaction^38^, and the No-runlength constraint can avoid consecutive identical bases. Homopolymers can make the sequence difficult to synthesize and increase the probability of misreading in the process of sequencing. The mathematical expression of the above constraints is shown in Eqs. (1–4), where the symbol meanings are kept consistent with the original text. The Hamming distance constraint is introduced as follows.

1 $$H\left( {x,y} \right) = \mathop {\sum}\limits_{i = 1}^n {h\left( {x_i,y_i} \right)} ,h\left( {x_i,y_i} \right) = \left\{ \begin{array}{l}0,x_i = y_i\\ 1,x_i \,\ne\, y_i\end{array} \right.$$

where *x* and *y* are a pair of DNA sequences. The Hamming distance is defined as *H (x, y)* ≥ *d*, and *H (x, y)* represents the number of different positions of *x* and *y*. The store edit distance constraint is shown below.

2 $$SE\left( {a_i} \right) = \mathop {{\min }}\limits_{1 \le j \le n,j \ne i} \left\{ {E\left( {a_i,b_j} \right)} \right\} \ge d$$

where *a, b* are DNA code words of length *n*, and *E(a, b)* is defined as the storage edit distance between *a* and *b*. *SE (a*_*i*_*)* defines the minimum *E (a*_*i*_*, b*_*j*_*)* in all DNA coding sets, which should not be greater than the element *d*. The MFE constraint is introduced as follows.

3 $$t = \frac{{\mathop {\sum}\limits_{i = 1}^n {\Delta M\left( {s_i,s_i^\prime } \right)} }}{n}$$

where *s* and *s’* are complementary DNA sequences, The Δ*M(s, s’)* denotes the value of MFE between *s, s’*, Given the threshold parameter *t*, the following constraints are given based on MFE, for all pairs of *s* in *S*, Δ*M(s,* *s’)* ≤ *t*, namely Δ*M(S)* = *max s* *∈* *S[ΔM(s,* *s’)]* ≤ *t*. Parameter t can be a constant or have other values.

The No-runlength constraint requires that there is *a* DNA sequence *L* (*l*_*1*_*, l*_*2*_*, l*_*3*_*…, l*_*n*_) of length *n* such that for any *i* satisfy formula (4):

4 $$\begin{array}{*{20}{c}} {l_{{{\mathrm{i}}}} \,\ne\, l_{{{{\mathrm{i + 1}}}}}} & {{{{\mathrm{i}}}} \in \left[ {{{{\mathrm{1,}}}}\,n{{{\mathrm{ - 1}}}}} \right]} \end{array}$$

### Base balance degree constraint

**Fig. 7:**
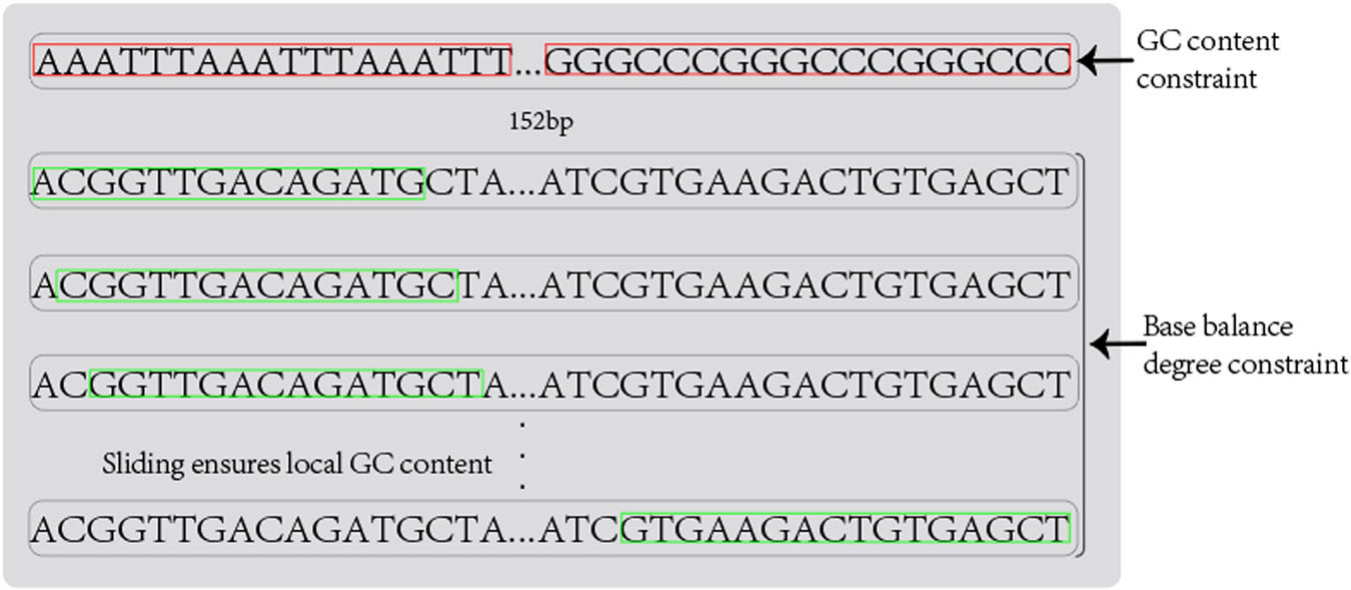
The difference between base balance degree constraint and GC content constraint.

To reduce the higher DNA sequencing coverage when low error rate reads are performed on the information stored in DNA, this section proposes the base balance degree constraint applied in the payload encoding phase of the adaptive encoding scheme. Although GC content and homopolymer constraints can be applied to fountain code coding, the base balance degree constraint has unique advantages over GC content constraint. As shown in Fig. 7, in the case of a long DNA sequence, the encoding constraint GC content is no longer applicable. The amount of G and C in a sequence as a whole may be satisfactory, but the bases in a fragment are extremely unbalanced. But the base balance degree constraint overcomes this defect and satisfies the base balance of any DNA fragment. The sequence satisfying the constraint is obtained through a continuous loop during coding, which increases the computation and time, but it is worth it. DNA sequences with high base balance have a high diversity of bases. A diverse balanced library is important for generating high-quality sequencing data which can reduce the number of repeat sequencings and thus the coverage can be reduced. Base balance refers to the relative proportions of A, C, G, and T bases in each cycle of the run. Libraries with high base balance have approximately the same proportion of all four bases in each cycle throughout the sequencing process. When sequencing highly diverse libraries, the high-throughput sequencing system can maintain focus and easily register images to the cluster map, thus accomplishing the objective of delivering high-quality data^36^. The base equilibrium constraint is defined as the length in a DNA sequence *X* of length *l* such that for any *i ∈ (1, l-a)* there exists a subsequence $X_j\left( {i,a + i} \right)$ of DNA sequence *X* satisfying

5 $$\begin{array}{*{20}{c}} {0.45 \le GC\left( {X_j\left( {i,a + i} \right)} \right) \le 0.55} & {i \in \left( {1,l - a} \right)} \end{array}$$

In order to distinguish the difference between the base balance degree constraint and the conventional GC content constraint, a schematic diagram 7 is given. In the case of longer DNA sequence length, the coding constraint GC content is no longer applicable, because only the overall control of the base G and C content ratio, may appear that the overall GC content is satisfied, but there are extremely unbalanced bases in a certain segment. For example, in Fig. 7, the first sequence satisfies the overall GC content, but because the sequence is long, poor base distribution in the red box is possible. According to the Illumina second-generation sequencing manual^36^, this base distribution will make the light signal difficult to identify. That’s because Illumina sequencers are sophisticated optical instruments that rely on changes in light signals to identify bases. Balanced bases show roughly equal signals in each sequencing channel, while unbalanced bases show that there may be more of one signal in the channel, and thus less of the other bases^36^. When all clusters provide signals primarily in one channel, the instrument may have difficulty identifying the location of the cluster and making base calls. The optical signal that is difficult to identify will reduce the accuracy of sequencing data. In this case, if we want to obtain high-quality sequencing data, we need to increase the sequencing coverage. But this increases the cost of time and consumables.

High base balance is critical for optimal run performance and high-quality data generation, and it is particularly important in the first 25 cycles of the sequencing run, as this is the time for clustering through filters, phase/pre-phase, and color matrix correction calculations. For platforms with non-modal flow cells such as MiniSeq™, MiSeq, NextSeq™500/550, and HiSeq™1000/2500, base balance is important during template generation. Illumina reported thumbnails of balanced and unbalanced libraries in a MiSeq™ sequencing cycle and showed approximately equal signals in each lane^36^. When all clusters provide signals primarily in one channel, it may be difficult for the instrument to identify cluster positions.

### Adaptive coding process

Existing DNA storage systems do not utilize bases sufficiently, and the storage density has some distance from the theoretical value. Moreover, the integrity between different coding positions is poor. Therefore, this paper proposes an adaptive coding scheme. The adaptive process is reflected in the adaptive generation of threshold values of non-payload coding constraints by payload bits. Since different positions of DNA sequences in DNA storage play different functions and have different error tolerance, different encoding methods are used at different encoding positions.

To further improve the storage density, the encoding method of fountain codes satisfying GC content, continuity, and base balance degree constraints is used in the encoding of the payload, and RS codes and LT codes are used as internal codes. The coding process first redundantly evaluates the polynomial at multiple points and then transmits or stores it. When the receiver correctly receives enough points, it can recover the original polynomial even if many noise points are interfering with the received polynomial. Formally, the set of code words R for RS codes are defined as follows:

6 $${{{\mathrm{R}}}} = \left\{ {\left. {\left( {p\left( {a_1} \right),\,p\left( {a_2} \right), \ldots ,p\left( {a_n} \right)} \right)} \right|\,p\,{{{\mathrm{is}}}}\,{{{\mathrm{a}}}}\,{{{\mathrm{poly}}}}\,{{{\mathrm{over}}}}\,\,F\,{{{\mathrm{of}}}}\,{{{\mathrm{degree}}}}\, < k} \right\}$$

LT codes are the first practical fountain codes that come closest to perfect erasure correcting codes. LT encoding and decoding are performed as in Eqs. (7) and (8).

7 $$\left( {M_1 \oplus M_2 \ldots \oplus M_d} \right),1 \ll d \ll n$$

where $M_1,M_2 \ldots ,M_d$ is the selected group *d* out of the total *n* groups of packets.

8 $$\begin{array}{l}\left( {M_1 \oplus \ldots \oplus M_d} \right) \oplus \left( {M_1 \oplus \ldots \oplus M_{k - 1} \oplus M_{k + 1} \oplus \ldots \oplus M_d} \right)\\ \,= M_1 \oplus M_1 \oplus \ldots \oplus M_{k - 1} \oplus M_{k - 1} \oplus M_k \oplus M_{k + 1} \oplus M_{k + 1} \oplus \ldots \oplus M_d \oplus M_d\\ \,= 0 \oplus \ldots \oplus 0 \oplus M_k \oplus 0 \oplus \ldots \oplus 0\\ \,= M_k\end{array}$$

where *n* and *k* satisfy *1* ≤ *k* ≤ *n* ≤ *|* *F* | , and *R* is an [*n, k, n-k* + *1*] code, which is a linear code of length *n*, dimension *k*, and minimum Hamming distance *n-k* + *1* in *F*.

Similarly, to improve the coding quality, imposing constraints on the constructed non-payload can ensure the stability of the non-payload during storage and reduce the probability of error occurrence while also reducing the coverage. The constrained encoding of the non-payload can be approximated as a multi-objective combinatorial optimization problem and constructed using heuristic class algorithms. Therefore, a combination of a heuristic class algorithm and a combinatorial constraint construction scheme is used in the encoding process of non-payload. All subsets in the set *S* of non-payload encodings need to satisfy the given encoding constraints such that any two encodings *c*_*i*_ and *c*_*j*_ in the subset *C* satisfy:

9 $$\tau \left( {c_i,c_j} \right) \ge k$$

here, *k* is a specific positive integer, and the symbol τ denotes the encoding constraints that should be satisfied by the two encodings *c*_*i*_ and *c*_*j*_, such as Hamming distance, storage editing distance, GC content, and MFE constraints.

**Fig. 8:**
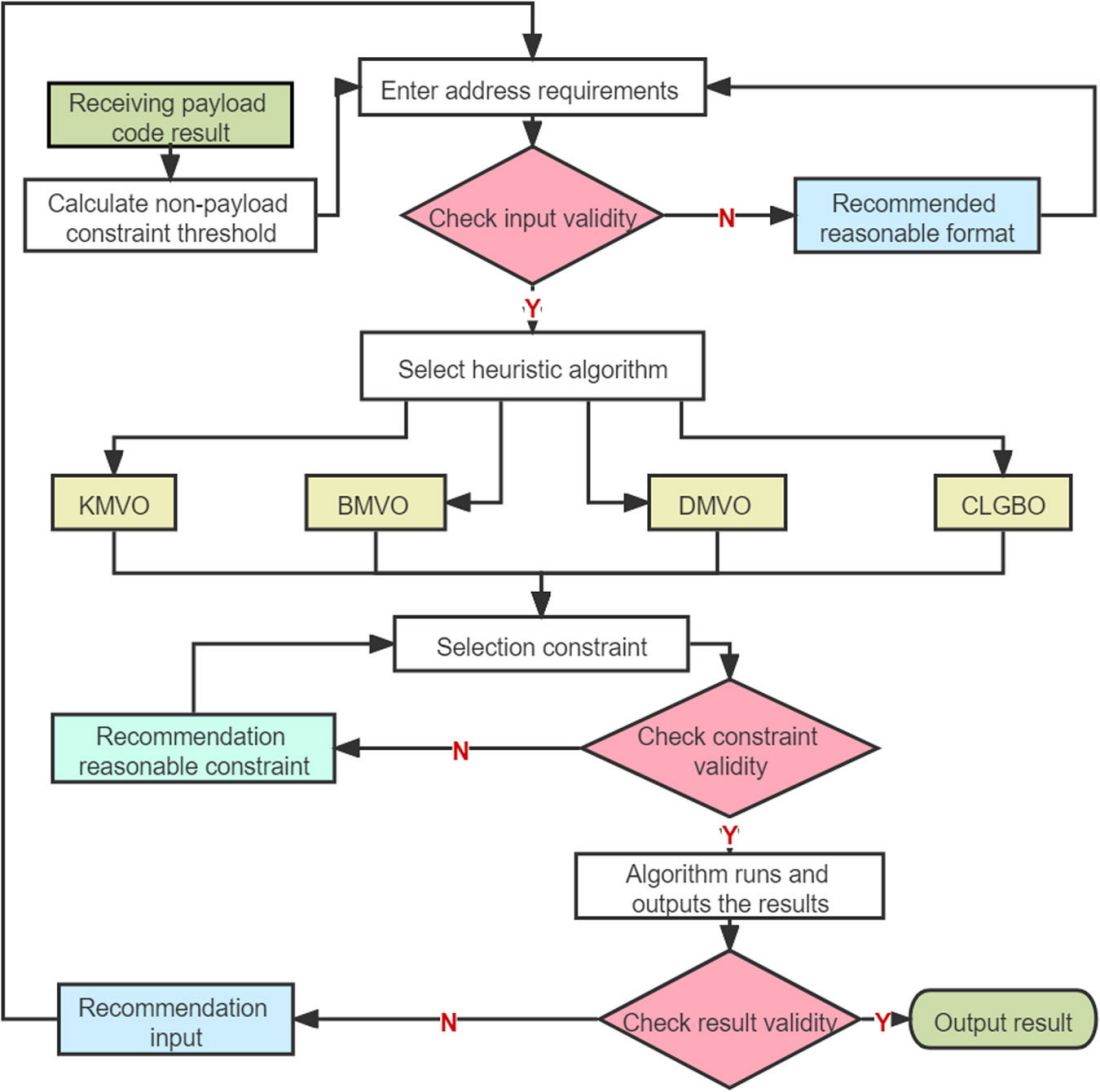
Constraints and algorithms are optional threshold adaptive non-payload coding algorithms.

In the process of non-payload coding, the coding constraint threshold to be satisfied is adaptively generated by payload coding results, such as GC content, continuous base, etc. For the problem that different locations of DNA sequences in existing storage systems exercise different functions and the coupling between each different encoding location is poor, adaptive generation of non-payload encoding thresholds is used to strengthen the connection between different encoding locations. The coding threshold adaptive method can adaptively generate the threshold in the non-payload combination constraint by the result of payload, as shown in Fig. 8. Moreover, reducing the coordination error rate in DNA data storage by adaptively generating coding thresholds can improve the throughput and reduce the sequencing coverage. Previous work has effectively advanced coding at various locations but lacks a unified treatment of coordination errors from a system level. Non-payload constraint threshold adaptive coding improves the ability to handle coordination errors. For example, GC content works best at around 50% in DNA synthesis and sequencing, but previous work has focused only on partially encoded GC content. In the adaptive coding algorithm for non-payload bits proposed in this paper, the GC content threshold during non-payload coding can be set in the DNA storage system based on the GC content encoded in the payload bits, which is calculated as in Eq. (10). The table of lower bounds for the set of non-payload encoding under different constraints is given in Supplementary Table 4.

10 $$GC(nopay) = \frac{{50\% (length(nopay) + length(pay)) - pay_{GC}}}{{length(nopay)}}$$

where nopay and pay represent non-payload and payload, respectively; *GC (nopay)* is the threshold of the GC content of the non-payload that needs to be generated adaptively; *pay*_*GC*_ is the number of bases of G and C in the payload.

The process of adaptive generation of thresholds for non-payload encoding constraints is as follows.

Step 1: receive the set of encoded payload codes completed by encoding.

Step 2: calculate eigenvalue such as GC content and continuity of the payload encoding set.

Step 3: calculate the threshold value for generating the non-payload GC content, continuity threshold, and whether uncorrelation in the address constraint is required.

Step 4: input the number of address bits needed to encode the set, and determine whether the current threshold condition exceeds the lower bound of the non-payload encoding set.

Step 5: return to step 4 if it exceeds; otherwise, to step 6.

Step 6: continue the next encoding process with the current non-payload encoding threshold as in Fig. 8.

**Fig. 9:**
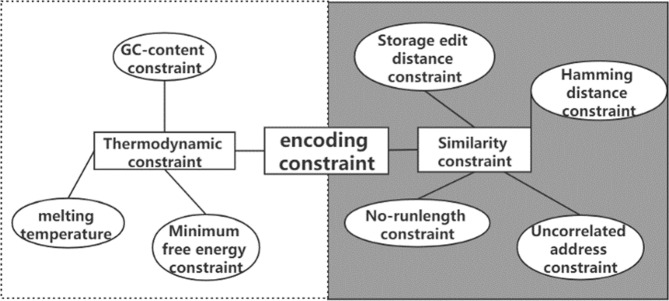
Classification and overlap of coding constraints.

The adaptive generation of non-payload encoding thresholds for payload bits enables a strong coupling between the non-payload and payload, which can reduce the probability of errors in DNA assembly. In Fig. 5, the Gibson assembly process is shown, in which the address and data bits need to be spliced, and it is crucial to ensure the stability of the assembly process as the DNA single-strand is exposed during the digestion of the 5’ end by the nucleic acid exonuclease, while the strong coupling between the non-payload and payload reduces the address–memory crosstalk generation. To use bases more rationally, the non-payload coding scheme supports heuristic class algorithms and constraints for selective use, which are reasonably chosen for different storage conditions, different storage contents, different experimental environments, and different storage overheads. The currently supported heuristic class algorithms are KMVO^33^, DMVO^32^, BMVO^38^, and CLGBO^41^, and the supported constraints are Hamming distance constraint, storage edit distance constraint, GC content constraint, no-runlength constraint, and minimum value-free energy constraint. K-means Multi-Verse Optimizer (KMVO) algorithm^33^ is an improvement of the Multi-Verse Optimizer (MVO) algorithm by K-means clustering. Wang et al.^32^ proposed Damping Multi-Verse Optimizer (DMVO) algorithm on the basis of MVO algorithm by adding a disturbance factor. The Brown Multi-Verse Optimizer (BMVO) algorithm was obtained by adding Brownian motion and single-line method to MVO algorithm, and the CLGBO algorithm^41^ improved the Gradient-based optimizer (GBO) algorithm by adopting Cauchy mutation operator and Levy strategy. The overlap of candidate solutions under different constraints is shown in Fig. 9. For example, the GC content constraint and TM constraint imply a lower utilization of bases. It is necessary to classify and use constraints selectively, and different combinations of constraints can be chosen according to different requirements (real problems). The computational complexity can be reduced, and the base utilization can be improved. By the way, a combination constraint can be any combination of constraints in the diagram.

### Independent random access

**Fig. 10:**
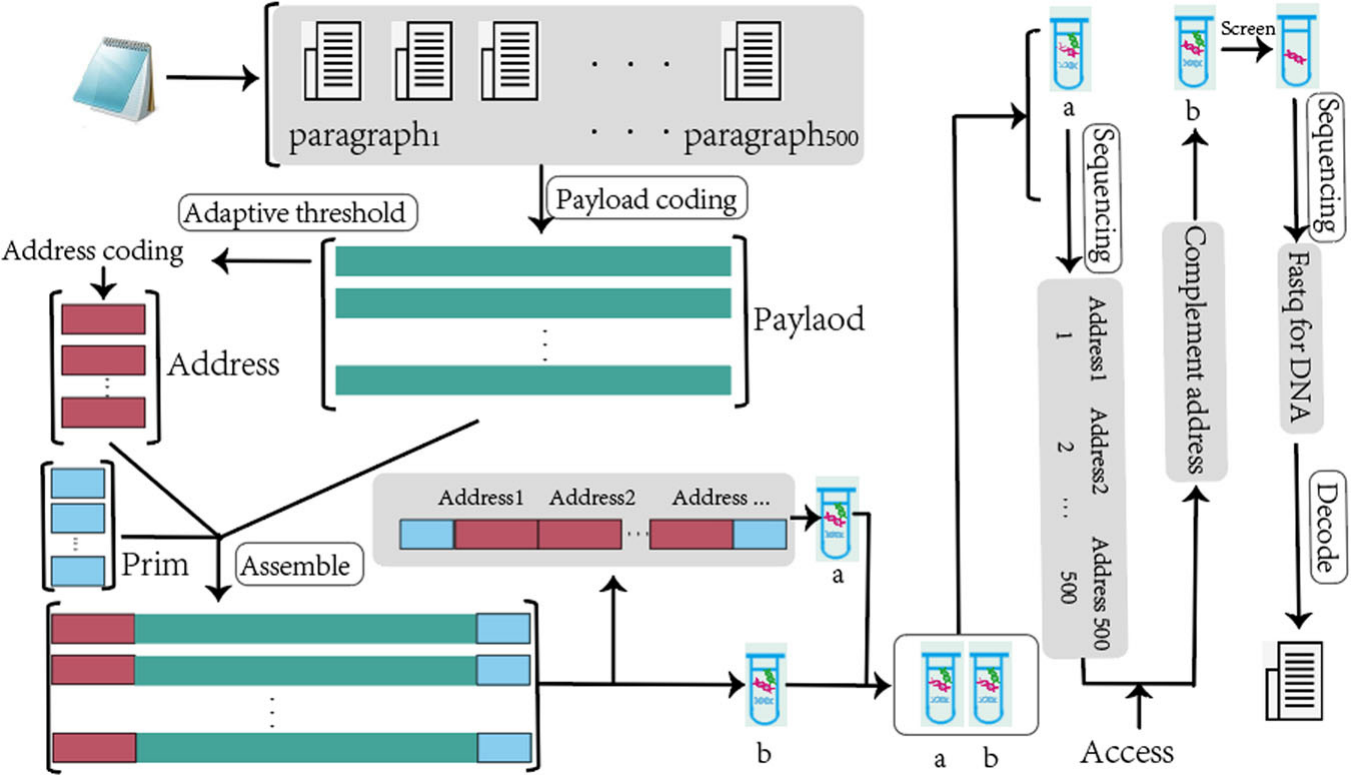
Independent random storage that requires no additional storage.

In order to better illustrate the performance of the adaptive DNA storage system proposed in this paper, an independent random storage test is performed in this section. Storage samples are excerpts from Harry Potter. It is worth mentioning that in this section, all the information needed to be used in random access is stored in DNA. Although this may reduce the storage density, it is necessary for random access DNA storage systems, the specific process is shown in Fig. 10.

In this strictly random access experiment, the first 500 segments of Harry Potter 1 are selected for storage, and the strategy of encoding according to different characteristics is used to assemble the DNA sequence after adaptive coding, and the address bit information is assembled into additional DNA sequences, which are stored in DNA pool an (index pool). The complete DNA sequence containing address bits, primers, and payload bits is stored in DNA pool b, also known as the archive pool. When random access to data is required, all index pools are sequenced first, and the results are decoded and restored into indexes. Then, the access request is synthesized into the complementary sequence of address bits, and the DNA sequence to be retrieved is separated by PCR amplification and purification in the archive pool. Finally, the separated DNA sequence is sequenced and decoded into the original information to obtain the access content.

## Supplementary information

Supplementary document

## Data Availability

All data are available at the following code repository: https://github.com/caobencs/Adaptive-DNA-Storage with the MIT license. Supplementary data are available at Npj online.

## Code availability

Code is available at the following code repository: https://github.com/caobencs/Adaptive-DNA-Storage with the MIT license.

## References

[1] Davis J (1996). Microvenus. Art. J..

[2] Bancroft C, Bowler T, Bloom B, Clelland C (2001). Long-Term Storage of Information in DNA. Science.

[3] Church GM, Gao Y, Kosuri S (2012). Next-generation digital information storage in DNA. Science.

[4] Goldman N (2013). Towards practical, high-capacity, low-maintenance information storage in synthesized DNA. Nature.

[5] Yazdi S, Gabrys R, Milenkovic O (2017). Portable and error-free DNA-based data storage. Sci. Rep..

[6] Organick L (2018). Random access in large-scale DNA data storage. Nat. Biotechnol..

[7] Lee HH, Kalhor R, Goela N, Bolot J, Church GM (2019). Terminator-free template-independent enzymatic DNA synthesis for digital information storage. Nat. Commun..

[8] Bee C (2021). Molecular-level similarity search brings computing to DNA data storage. Nat. Commun..

[9] Banal JL (2021). Random access DNA memory using Boolean search in an archival file storage system. Nat. Mater..

[10] Tavella F (2021). DNA molecular storage system: transferring digitally encoded information through bacterial nanonetworks. IEEE Trans. Emerg. Top. Comput..

[11] Bhattarai-Kline S, Lear SK, Shipman SL (2021). One-step data storage in cellular DNA. Nat. Chem. Biol..

[12] Yin, Q., Zheng, Y., Wang, B., & Zhang, Q. Design of Constraint Coding Sets for Archive DNA Storage. *IEEE/ACM Transactions on Computational Biology and Bioinformatics*, pp. 1–1, 2021. 10.1109/TCBB.2021.3127271. 10.1109/TCBB.2021.3127271 34762590

[13] Bornholt J (2017). Toward a dna-based archival storage system. IEEE Micro..

[14] Grass RN, Heckel R, Puddu M, Paunescu D, Stark WJ (2015). Robust chemical preservation of digital information on DNA in silica with error-correcting codes. Angew. Chem. Int. Ed..

[15] Bornhol J (2016). A DNA-based archival storage system. Acm Sigplan Not..

[16] Erlich Y, Zielinski D (2017). DNA Fountain enables a robust and efficient storage architecture. Science.

[17] Jeong, J. et al. Cooperative sequence clustering and decoding for DNA storage system with fountain codes. *Bioinformatics,* btab246 (Oxford, England, 2021). 10.1093/bioinformatics/btab246 33904574

[18] Anavy L, Vaknin I, Atar O, Amit R, Yakhini Z (2019). Data storage in DNA with fewer synthesis cycles using composite DNA letters. Nat. Biotechnol..

[19] Immink KAS, Cai K (2018). Design of capacity-approaching constrained codes for DNA-based storage systems. IEEE Commun. Lett..

[20] Yazdi S, Kiah HM, Gabrys R, Milenkovic O (2018). Mutually uncorrelated primers for DNA-based data storage. IEEE Trans. Inf. Theory.

[21] Song WT, Cai K, Zhang M, Yuen C (2018). Codes with run-length and GC-content constraints for DNA-based data storage. IEEE Commun. Lett..

[22] Wang YX, Noor-A-Rahim M, Gunawan E, Guan YL, Poh CL (2019). Construction of bio-constrained code for DNA data storage. IEEE Commun. Lett..

[23] Press WH, Hawkins JA, Jones SK, Schaub JM, Finkelstein IJ (2020). HEDGES error-correcting code for DNA storage corrects indels and allows sequence constraints. Proc. Natl Acad. Sci. USA.

[24] Lenz A, Siegel PH, Wachter-Zeh A, Yaakobi E (2020). Coding over sets for DNA storage. IEEE Trans. Inf. Theory.

[25] Fei, P., & Wang, Z. LDPC codes for portable DNA storage, in international symposium on information theory, France, 2019, pp. 76–80.

[26] Choi, Y., et al. DNA Micro-disks for the management of DNA-based data storage with index and write-once-read-many (WORM) memory features. *Adv. Mater.* **32**, 2001249 (2020). 10.1002/adma.202001249 32725925

[27] Zhu J, Ermann N, Chen K, Keyser UF (2021). Image encoding using multi-level DNA barcodes with nanopore readout. Small.

[28] Song L, Zeng A (2018). Orthogonal information encoding in living cells with high error-tolerance, safety, and fidelity. ACS Synth. Biol..

[29] Organick L (2018). Random access in large-scale DNA data storage. Nat. Biotechnol..

[30] Meiser LC (2020). Reading and writing digital data in DNA. Nat. Protoc..

[31] Ceze L, Nivala J, Strauss K (2019). Molecular digital data storage using DNA. Nat. Rev. Genet..

[32] Cao B (2022). Designing uncorrelated address constrain for DNA storage by DMVO algorithm. IEEE/ACM Trans. Comput. Biol. Bioinformatics..

[33] Cao B, Zhao S, Li X, Wang B (2020). K-means multi-verse optimizer (KMVO) algorithm to construct DNA storage codes. IEEE Access.

[34] Wang, Y. X. et al. High capacity DNA data storage with variable-length Oligonucleotides using repeat accumulate code and hybrid mapping. *J. Biol. Eng.* **13**, 89 (2019). 10.1186/s13036-019-0211-2 PMC6868767 31832092

[35] Baum EB (1995). Building an associative memory vastly larger than the brain. Science.

[36] illumina. https://support.illumina.com/bulletins/2016/07/what-is-nucleotide-diversity-and-why-is-it-important.html.

[37] Ross MG (2013). Characterizing and measuring bias in sequence data. Genome Biol..

[38] Wu J, Zheng Y, Wang B, Zhang Q (2022). Enhancing physical and thermodynamic properties of DNA storage sets with end-constraint. IEEE Trans. Nanobiosci..

[39] Huang W, Li L, Myers JR, Marth GT (2011). ART: a next-generation sequencing read simulator. Bioinformatics.

[40] Cao B (2021). Minimum free energy coding for DNA storage. IEEE Trans. Nanobiosci..

[41] Zheng, Y., Wu, J., & Wang, B. CLGBO: an algorithm for constructing highly robust coding sets for DNA storage. *Front. Genet.* **12**, 644945 (2021). 10.3389/fgene.2021.644945 PMC8129200 34017354

[42] Bornholt, J. et al. Toward A DNA-based archival storage system. *IEEE MICRO*, 37 pp. 98–104 (2016).

[43] Blawat M (2016). Forward error correction for DNA data storage. Procedia Comput. Sci..

[44] Yazdi, S., Yuan, Y.B., Ma, J., Zhao, H.M., & Milenkovic, O. A rewritable, random-access DNA-based storage system. *Sci. Rep.* **5**, 14138 (2015). 10.1038/srep14138 PMC4585656 26382652

