# Supplementary material for: Adaptive coding for DNA storage with high storage density and low coverage
Source: NPJ Syst Biol Appl. 2022 Jul 4;8:23. doi: 10.1038/s41540-022-00233-w (PMC9253015; doi:10.1038/s41540-022-00233-w)
Supplement: Supplementary file 1 — Supplementary document [file 41540_2022_233_MOESM1_ESM.pdf]

# Adaptive coding for DNA storage with high storage density and low coverage

**Supplementary document 1**

DNA subsequences with length of 100 were randomly extracted from each DNA sequence, and the length of each sequence was 100 base, that is, the length of each DNA sequence was 199 in Jeong's work, so 99 times were extracted from each DNA sequence. The variance of the ratio of bases A, T, G and C was calculated, and then the variance of the variance was calculated. The results in Table 1-3 are the sum, mean, and median of variances, used to reflect the base diversity of each DNA block.

**Supplementary document 2**

In the text, the coding results of the proposed cascading coding algorithm are compared with those of Jeong when the length is 137. In order to better illustrate the excellent performance of the adaptive coding algorithm, we also compare the results of different lengths under different constraints, and the abbreviations are consistent with those in the text.

**Supplementary document 3**

Jeong'work: Each oligo has length 199 nucleotides (nt) in total (26nt first primers+16nt seed + 128nt payload + 8nt RS code+21nt end primers)

513.6KB=513.6x8x1024=4207411.2bit

152x18000=2736000nt 4207411.2bit/2736000nt=1.53bit/nt

199x18000=3582000nt 4207411.2bit/3582000nt=1.17bit/nt

Ourwork: Each oligo has length 172 nucleotides (nt) in total (10nt addrsss+16nt seed + 128nt payload + 8nt RS code+15nt end primers)

480kb=480x8x1024= 3932160bit

162x17200=2786400nt 3932160bit/2786400nt =1.41bit/nt

177x17200= 3044400nt 3932160bit/ 3044400nt=1.29bit/nt

Every time a DNA sequence is generated in an independent random storage experiment, an additional address bit needs to be stored, so the storage density of the independent random storage experiment is 1.22bit/nt. Calculations of other data refer to the work of Erlich and Ceze et al[1, 2]., which will not be described here.

**Supplementary document 4**

Table 1 gives the maximum value of non-payload constructed by heuristic algorithm under EGNA, EGNAM, HGNN and HGN, with data from Wang et al.[4-6]. According to the threshold value, the size of DNA pool in DNA storage can be reasonably divided. When the required non-payload exceeds the maximum under the current length, consideration is given to increasing the length of the non-payload.

**Supplementary tables 1.** Comparison of naked single strand MFE and TM in length 580.

| | MFEAve | MFEMAX | MFEMIN | TM variance |
|---|---|---|---|---|
| Jeong[3] | -11.9497 | -4.9 | -28.5 | 2.2797 |
| HGN | -14.5088 | -3.4 | -28.7 | 1.9737 |

**Supplementary tables 2.** Comparison of naked single strand MFE and TM in length 157.

| | MFEAve | MFEMAX | MFEMIN | TM variance |
|---|---|---|---|---|
| Jeong[3] | -11.4325 | -4.9 | -26.3 | 2.28 |
| HGNN | -14.4771 | -4.8 | -31.1 | 2.0177 |

**Supplementary tables 3.** Comparison of naked single strand MFE and TM in length 250.

| | MFEAve | MFEMAX | MFEMIN | TM variance |
|---|---|---|---|---|
| Jeong[3] | -11.5732 | -4.9 | -26.3 | 2.2879 |
| EGNAM | -14.3096 | -4.1 | -27.1 | 1.9859 |

**Supplementary tables 4.** The lower limit of the non-payload code set under four combinatorial constraints

| n\d | 3 | 4 | 5 | 6 | 7 | 8 | 9 | |
|---|---|---|---|---|---|---|---|---|
| 4 | 6 | — | | | | | | EGNA |
| | 4 | — | | | | | | EGNAM |
| | — | — | | | | | | HGNN |
| | 6 | 2 | | | | | | HGN |
| 5 | 12 | 5 | — | | | | | EGNA |
| | 7 | 2 | — | | | | | EGNAM |
| | — | — | — | | | | | HGNN |
| | 15 | 3 | 1 | | | | | HGN |
| 6 | 30 | 11 | 4 | — | | | | EGNA |
| | 16 | 7 | 2 | — | | | | EGNAM |
| | 51 | 22 | 8 | — | | | | HGNN |
| | 43 | 16 | 4 | 2 | | | | HGN |
| 7 | 53 | 19 | 6 | — | — | | | EGNA |
| | 28 | 11 | 3 | — | — | | | EGNAM |
| | 113 | 42 | 15 | 6 | — | | | HGNN |
| | 131 | 34 | 11 | 2 | 1 | | | HGN |
| 8 | 101 | 38 | 12 | 5 | 3 | — | | EGNA |
| | 48 | 21 | 9 | 3 | — | — | | EGNAM |
| | 319 | 105 | 35 | 15 | 5 | — | | HGNN |
| | 437 | 102 | 26 | 11 | 2 | 2 | | HGN |
| 9 | 167 | 61 | 19 | 7 | 3 | 2 | — | EGNA |
| | 93 | 33 | 12 | 5 | 2 | — | — | EGNAM |
| | 635 | 206 | 66 | 25 | 10 | 5 | — | HGNN |
| | 1331 | 280 | 65 | 19 | 8 | 2 | 1 | HGN |
| 10 | 250 | 110 | 34 | 11 | 5 | 3 | 2 | EGNA |
| | 137 | 62 | 19 | 6 | 3 | — | — | EGNAM |
| | 1634 | 518 | 157 | 56 | 21 | 10 | 4 | HGNN |
| | 4490 | 857 | 179 | 55 | 16 | 8 | 2 | HGN |

Reference


[1] L. Ceze, J. Nivala, and K. Strauss, “Molecular digital data storage using DNA,” *Nature Reviews Genetics,* vol. 20, no. 8, pp. 456-466, Aug, 2019.

[2] Y. Erlich, and D. Zielinski, “DNA Fountain enables a robust and efficient storage architecture,” *Science,* vol. 355, no. 6328, pp. 950-953, Mar, 2017.

[3] J. Jeong, S.-J. Park, J.-W. Kim, J.-S. No, H. H. Jeon, J. W. Lee, A. No, S. Kim, and H. Park, “Cooperative Sequence Clustering and Decoding for DNA Storage System with Fountain Codes,” *Bioinformatics (Oxford, England)*, 2021 Apr 27 (Epub 2021 Apr, 2021.

[4] B. Cao, X. Zhang, J. Wu, B. Wang, and Q. Zhang, “ Minimum free energy coding for DNA storage,” *IEEE Transactions on NanoBioscience,* vol. 2, pp. 212-222, 2021.

[5] B. Cao, X. Li, X. Zhang, B. Wang, Q. Zhang, and X. Wei, “Designing Uncorrelated Address Constrain for DNA Storage by DMVO Algorithm,” *IEEE/ACM Transactions on Computational Biology and Bioinformatics*, 2020.

[6] Yanfen Zheng, Jieqiong Wu , and Bin Wang, “CLGBO: An algorithm for constructing highly robust coding sets for DNA storage,” *frontiers in Genetics*, 2021.
